# Supplementary material for: Postoperative Hormone Replacement Therapy and Survival in Women with Ovarian Cancer
Source: Cancers (Basel). 2022 Jun 23;14(13):3090. doi: 10.3390/cancers14133090 (PMC9265037; doi:10.3390/cancers14133090)
Supplement: Supplementary file 1 [file cancers-14-03090-s001.zip › cancers-1723755-supplementary.pdf]

cancers

MDPI

Article

Postoperative Hormone Replacement Therapy and Survival in Women with Ovarian Cancer

Eunjeong Ji, Kidong Kim, Banghyun Lee, Sung Ook Hwang, Hee Joong Lee, Kyungjin Lee, Minkyung Lee and Yong Beom Kim

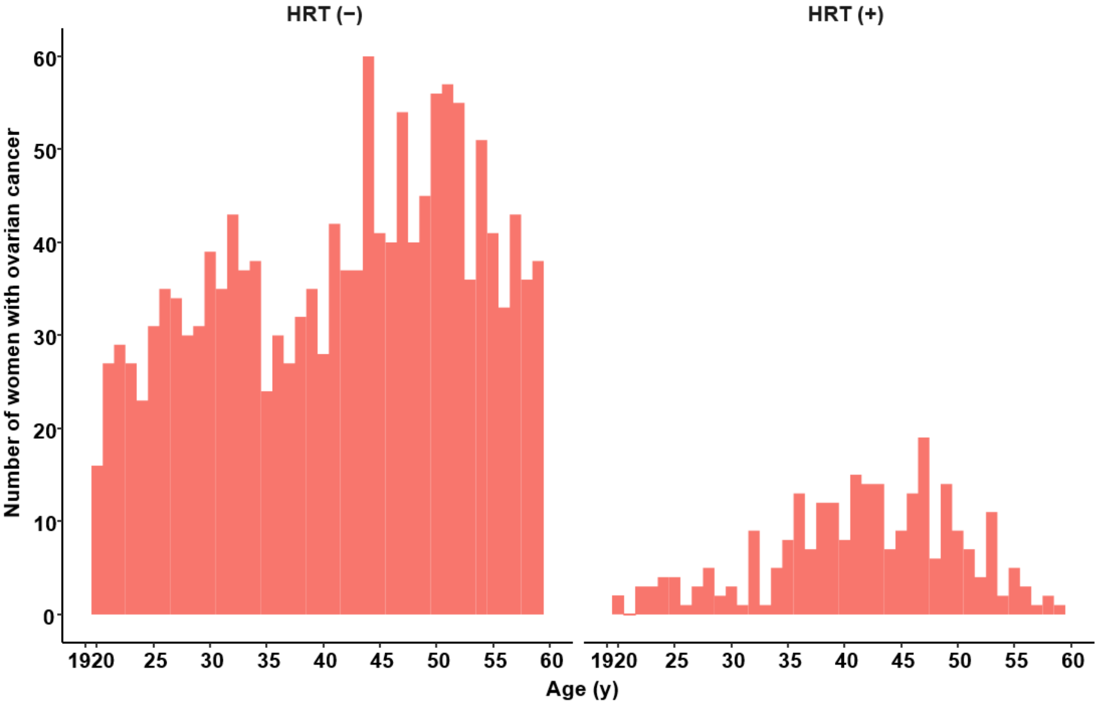

Figure S1. Age distributions of women with ovarian cancer according to the receipt of HRT.

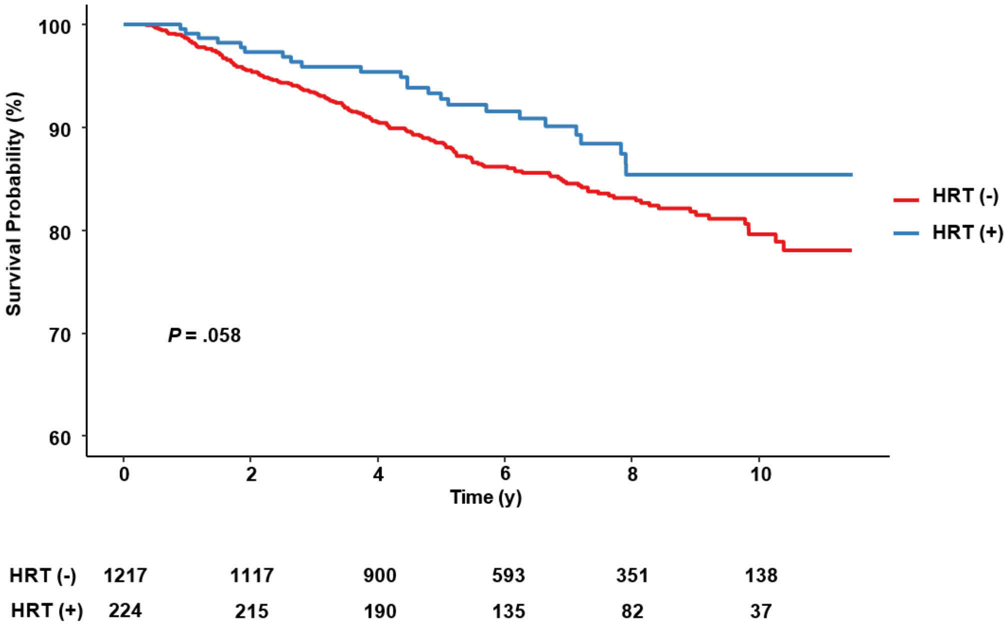

A

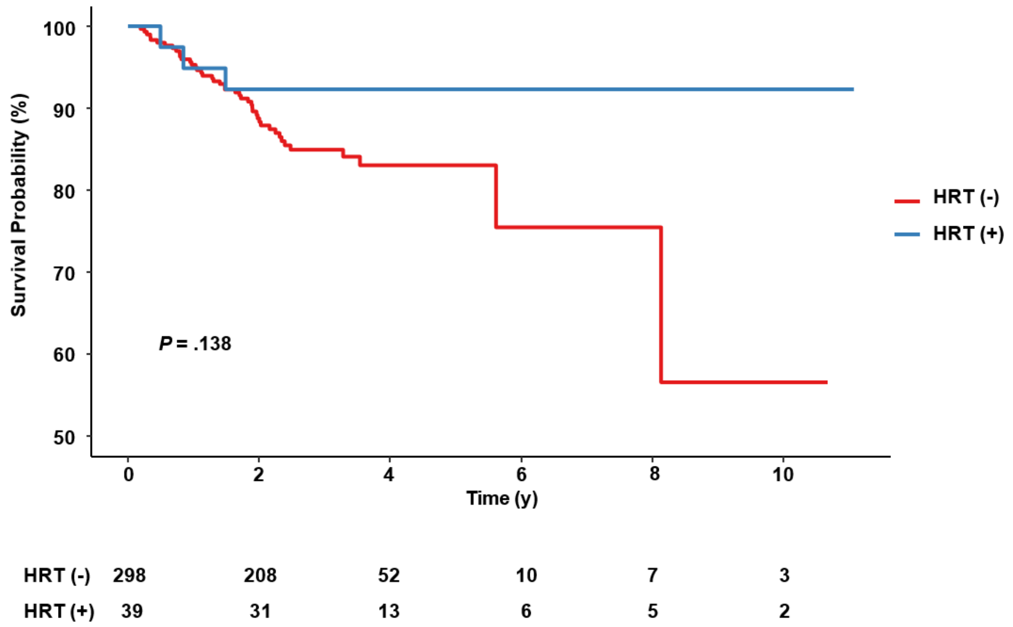
Survival Probability (%)
100
90
80
70
60
50
P = .138
0
2
4
6
8
10
Time (y)
HRT (-)
HRT (+)
HRT (-) 298 208 52 10 7 3
HRT (+) 39 31 13 6 5 2

B

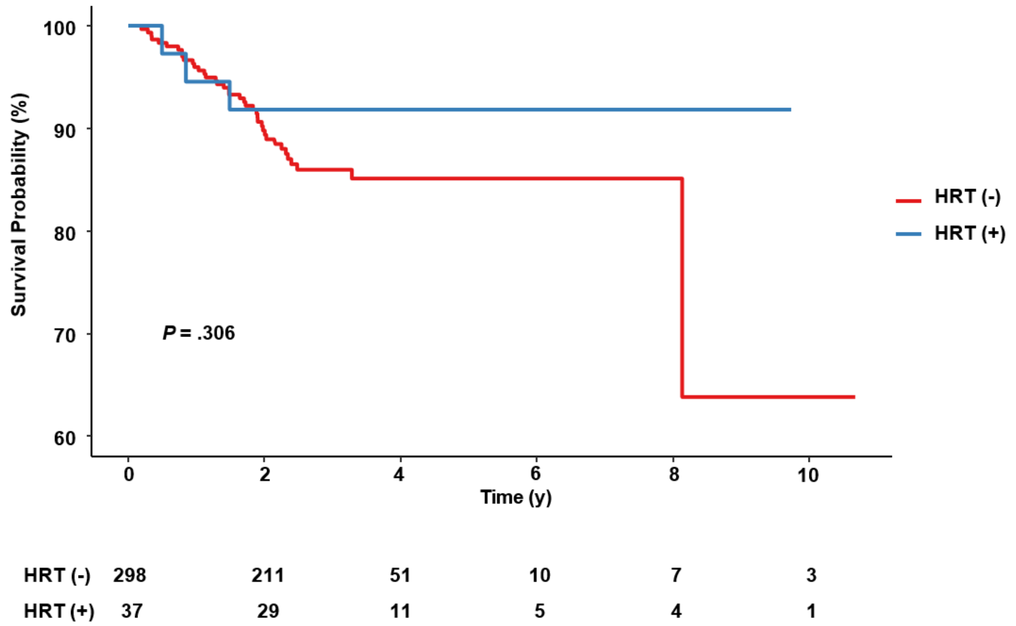
Survival Probability (%)
100
90
80
70
60
P = .306
0
2
4
6
8
10
Time (y)
HRT (-)
HRT (+)
HRT (-) 298 211 51 10 7 3
HRT (+) 37 29 11 5 4 1

C

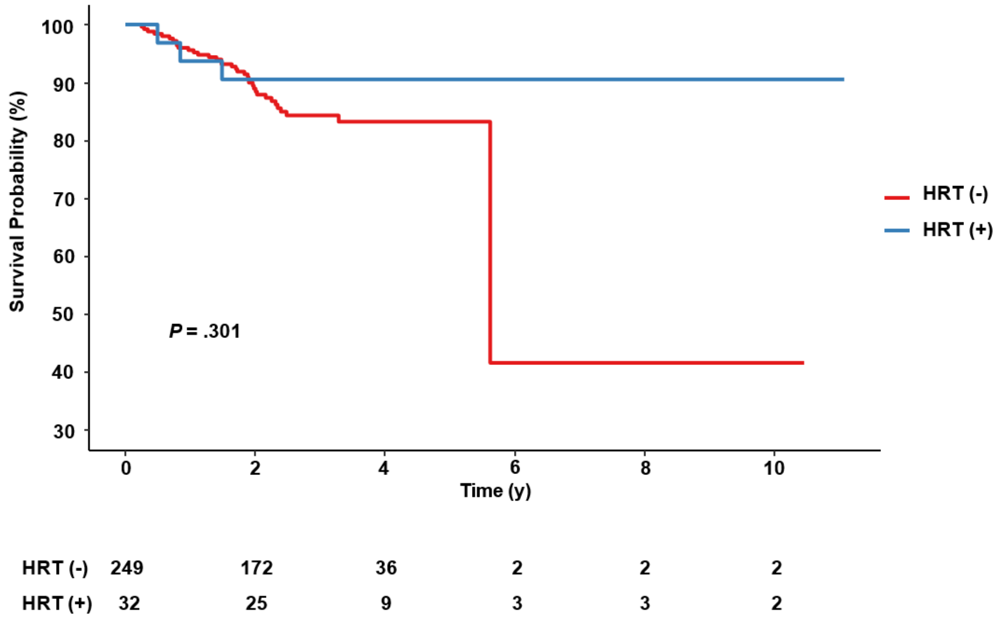

D

Figure S2. OS of women with ovarian cancer administered or not administered HRT according to surgical treatment types and chemotherapeutic agents. A. Women that received surgery alone, B. women that received surgery and adjuvant chemotherapy, C. women that received platinum-based chemotherapy, and D. women that received other chemotherapeutic agents.

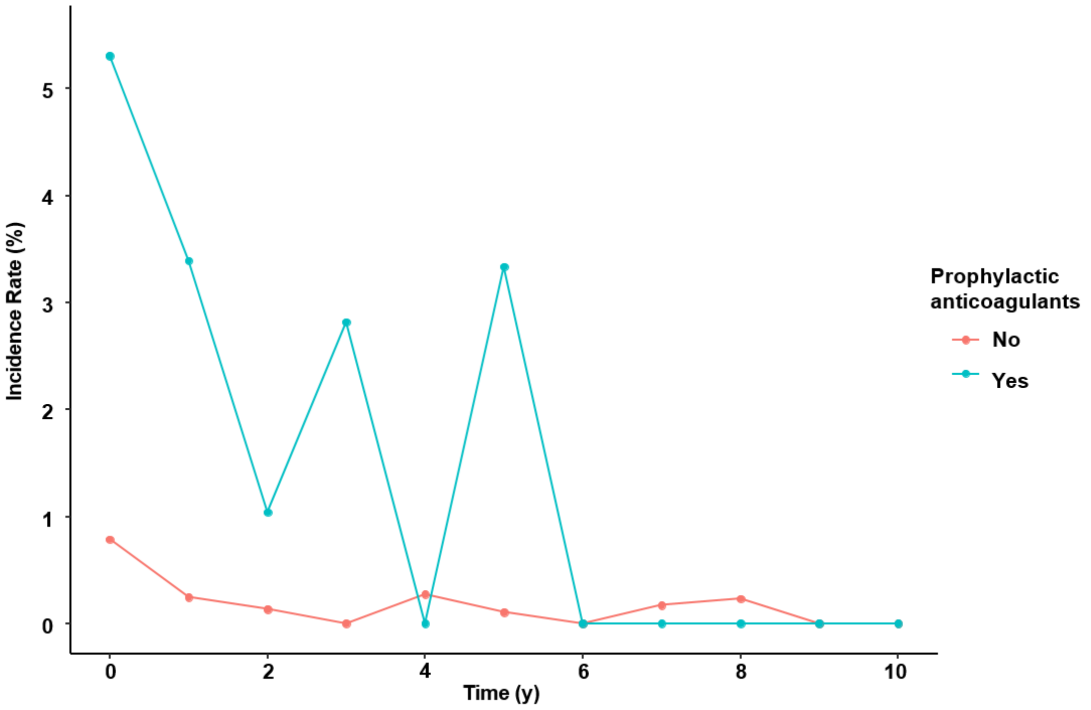

Figure S3. Incidences of VTE according to time after primary surgery in women with ovarian cancer administered or not administered prophylactic anticoagulants.

Table S1. Associations between risk factors and 5-year OS in women with ovarian cancer.

	Univariable analysis		Multivariable analysis [a,b]	
	HR (95% CI)	*P* value	HR (95% CI)	*P* value
Age, y	1.030 (1.016-1.044)	<.001	1.018 (1.004-1.033)	.01
SES				
Low SES	ref	.002	ref	.002
Mid- or high-SES	0.359 (0.190-0.679)		0.367 (0.195-0.691)	
CCI				
0	ref			
1	0.978 (0.705-1.355)	.892		
2	0.917 (0.562-1.497)	.729		
3	1.167 (0.543-2.512)	.692		
Over 4	0.872 (0.321-2.369)	.788		
Methods of primary surgery				
BSO, USO, or ovarian cystectomy	ref	.052		
Total hysterectomy ± BSO, USO, or ovarian cystectomy	1.466 (0.996-2.156)			
Other surgeries [c]				
(-)	ref	.008	ref	.121
(+)	1.576 (1.124-2.210)		1.312 (0.931-1.849)	
Types of primary surgery				
Surgery alone	ref			
Surgery + adjuvant chemotherapy	2.123 (1.505-2.994)	<.001 [b]		
Neoadjuvant chemotherapy + Surgery ± adjuvant chemotherapy	1.377 (0.084-22.504)	.822 [b]		
Postoperative VTE				
(-)	ref	<.001	ref	<.001
(+)	8.034 (5.092-12.675)		5.491 (3.388-8.901)	
Prophylactic anticoagulants				
(-)	ref	<.001	ref	<.001
(+)	4.276 (3.010-6.075)		2.656 (1.795-3.928)	
HRT				
(-)	ref	.021	ref	.029
(+)	0.564 (0.347-0.917)		0.583 (0.359-0.947)	

Abbreviations: BSO, bilateral salpingo-oophorectomy; CCI, Charlson comorbidity index; CI, confidence interval; HR, hazard ratio; HRT: hormone replacement therapy; SES, socioeconomic status; USO, unilateral salpingo-oophorectomy; VTE, venous thromboembolism. [a] Stratified cox proportional hazard model was used for multivariable model. [b] Firth penalized maximum-likelihood estimation was applied for this analysis. [c] Appendectomy, bowel resection, cholecystectomy, end-to-end ureteroureterostomy, pancreatectomy, partial gastrectomy, partial hepatectomy, pelvic and/or paraaortic lymph node dissection, splenectomy, stripping of other peritoneal surfaces, stripping of the diaphragm, ureteroneocystostomy.

Table S2. Incidences of postoperative VTE per 10,000 person-years according to use of prophylactic anticoagulants.

Prophylactic anticoagulants	Postoperative VTE				
	VTE case	Sum of person-years	IR per 10,000 person-years (95% CI)	IRR (95% CI)	*P* value
(-) (n = 1652)	25	9447.0	26.5 (17.1-39.1)		<.001
(+) (n = 132)	15	489.3	306.6 (171.6-505.6)	11.6 (5.7-22.8)	

Abbreviations: CI, confidence interval; IR, incidence rate; IRR, incidence rate ratio; VTE, venous thromboembolism.

Table S3. Postoperative VTE prophylaxis and treatment methods used in women with ovarian cancer..

	No. (%)	
	**Prophylaxis (n = 38)**	**Treatments (n = 49)**
UFH	11 (28.9)	22 (44.9)
LMWH	11 (28.9)	20 (40.8)
Fondaparinux	0 (0.0)	0 (0.0)
Warfarin	5 (13.2)	11 (22.5)
DOAC	2 (5.3)	30 (61.2)
Thrombectomy		0 (0.0)
Thromboplasty		0 (0.0)
Thrombolysis		1 (2.0)
IVC filter		23 (46.9)

Abbreviations: DOAC, direct oral anticoagulants; IVC, Inferior Vena Cava; LMWH, low molecular weight heparin; UFH, unfractionated heparin; VTE, venous thromboembolism. All values are expressed as number (%).

Table S4. Characteristics of women with ovarian cancer according to the administration of prophylactic anticoagulants.

**Characteristics**	No. (%)			***P* value**
	**Total**	**Prophylactic anticoagulants (-)**	**Prophylactic anticoagulants (+)**	
No. of women	1784 (100)	1652 (92.6)	132 (7.4)	
Age, mean (SD), y	41.0 (11.0)	40.8 (10.9)	44.7 (11.0)	<.001
SES				
Mid- or high-SES	1743 (97.7)	1616 (97.8)	127 (96.2)	.223 [a]
Low SES	41 (2.3)	36 (2.2)	5 (3.8)	
CCI				
0	943 (52.9)	889 (53.8)	54 (40.9)	<.001
1	549 (30.8)	512 (31.0)	37 (28.0)	
2	192 (10.8)	167 (10.1)	25 (18.9)	
3	59 (3.3)	51 (3.1)	8 (6.1)	
Over 4	41 (2.3)	33 (2.0)	8 (6.1)	
Year of cancer diagnosis				
2009	141 (7.9)	151 (8.4)	7 (5.2)	<.001
2010	168 (9.4)	187 (10.4)	3 (2.2)	
2011	143 (8.0)	159 (8.8)	4 (3.0)	
2012	173 (9.7)	182 (10.1)	8 (5.9)	
2013	157 (8.8)	166 (9.2)	5 (3.7)	
2014	200 (11.2)	208 (11.5)	8 (5.9)	
2015	193 (10.8)	193 (10.7)	18 (13.2)	
2016	189 (10.6)	163 (9.0)	32 (23.5)	
2017	205 (11.5)	192 (10.6)	31 (22.8)	
2018	215 (12.1)	203 (11.3)	20 (14.7)	
Methods of primary surgery				
BSO, USO, or ovarian cystectomy	1542 (86.4)	1439 (87.1)	103 (78.0)	.003

Total hysterectomy ± BSO, USO, or ovarian cystectomy	242 (13.6)	213 (12.9)	29 (22.0)	.003
Other surgeries [b]	290 (16.3)	251 (15.2)	39 (29.5)	<.001
Types of primary surgery				
Surgery alone	1441 (80.8)	1376 (83.3)	65 (49.2)	<.001
Surgery + adjuvant chemotherapy	337 (18.9)	274 (16.6)	63 (47.7)	<.001
Neoadjuvant chemotherapy + surgery ± adjuvant chemotherapy	6 (0.3)	2 (0.1)	4 (3.0)	<.001 [a]
Chemotherapy				
Platinum-based chemotherapy	335 (18.8)	274 (16.6)	61 (46.2)	<.001
Other agents	281 (15.8)	225 (13.6)	56 (42.4)	<.001
Bevacizumab ± any agents	7 (0.4)	4 (0.2)	3 (2.3)	.011 [a]
Postoperative VTE, n (%)				
(-)	1744	1627 (98.5)	117 (88.6)	<.001 [a]
(+)	40	25 (1.5)	15 (11.4)	
HRT				
Estrogen	196 (11.0)	184 (11.1)	12 (9.1)	.469
Estrogen + Progesterone	151 (8.5)	139 (8.4)	12 (9.1)	.788
Tibolone	161 (9.0)	150 (9.1)	11 (8.3)	
Duration of HRT, y				
≤ 0.5	43 (16.3)	38 (15.4)	5 (31.3)	
0.5-1	20 (7.6)	17 (6.9)	3 (18.8)	
1-2	47 (17.9)	46 (18.6)	1 (6.3)	
2-3	24 (9.1)	22 (8.9)	2 (12.5)	
3-4	31 (11.8)	28 (11.3)	3 (18.8)	
4-5	30 (11.4)	30 (12.1)	(0.0)	
> 5	68 (25.9)	66 (26.7)	2 (12.5)	
Time between primary surgery and VTE diagnosis, mean (SD), d	2034.3 (1083.0)	2088.7 (1078.1)	1354.0 (901.4)	

Abbreviations: BSO, bilateral salpingo-oophorectomy; CCI, Charlson comorbidity index; HRT: hormone replacement therapy; SD: standard deviation; SES, socioeconomic status; USO, unilateral salpingo-oophorectomy; VTE, venous thromboembolism. [a] The Fisher's exact test was used for this analysis. [b] Appendectomy, bowel resection, cholecystectomy, end-to-end ureteroureterostomy, pancreatectomy, partial gastrectomy, partial hepatectomy, pelvic and/or paraaortic lymph node dissection, splenectomy, stripping of other peritoneal surfaces, stripping of the diaphragm, ureteroneocystostomy.
